# Characteristics of solar-irradiance spectra from measurements, modeling, and theoretical approach

**DOI:** 10.1038/s41377-022-00750-7

**Published:** 2022-03-29

**Authors:** Gerard Thuillier, Ping Zhu, Martin Snow, Peng Zhang, Xin Ye

**Affiliations:** 1 Physikalisch-Meteorologisches Observatorium Davos World Radiation Centre (PMOD/WRC), Davos Dorf, Switzerland; 2 Changchun Institute of Optics, Fine Mechanics and Physics, Chinese Academy of Science, 3888 Dong Nanhu Road, Changchun, 130033 China; 3 Royal Observatory of Belgium, Av. Circulaire 3, 1180 Brussels, Belgium; 4 Laboratory for Atmospheric and Space Physics, University of Colorado Boulder, Boulder, CO 80309 USA; 5 South African National Space Agency (SANSA), Hospital Street, Hermanus, 7200 South Africa; 6 University of the Western Cape, Department of Physics and Astronomy, Robert Sobukwe Rd, Belville, Cape Town, 7535 South Africa; 7 National Satellite Meteorological Center, China Meteorological Administration, Beijing, 100081 China; 8 Innovation Center for FengYun Meteorological Satellite, China Meteorological Administration, Beijing, 100081 China

**Keywords:** Physics, Electronics, photonics and device physics

## Abstract

An accurate solar-irradiance spectrum is needed as an input to any planetary atmosphere or climate model. Depending on the spectral characteristics of the chosen model, uncertainties in the irradiance may introduce significant differences in atmospheric and climate predictions. This is why several solar spectral-irradiance data sets have been published during the last decade. They have been obtained by different methods: either measurements from a single instrument or a composite of different spectra, or they are theoretical or semi-empirical solar models. In this paper, these spectral datasets will be compared in terms of irradiance, power per spectral interval, their derived solar-atmosphere brightness temperature, and time series. Whatever the different sources of these spectra are, they generally agree to within their quoted accuracy. The solar-rotation effect simultaneously observed by SORCE and PREMOS–PICARD is accurately measured. The 11-year long-term variability remains a difficult task, given the weak activity of solar cycle 24 and long-term instrument aging.

## Introduction

Solar spectral irradiance (SSI) contains information characterizing the physical, chemical, thermal, and dynamical properties of the solar atmosphere. Furthermore, the total solar irradiance (TSI, formerly known as the solar constant) is an estimation of the total power radiated by the Sun.

Due to absorption by the Earth’s atmosphere, accurate SSI and TSI measurements must be made from space. Measurements from the ground introduce either missing spectral domains or very high uncertainties in retrieving absolute irradiances due to absorption by the Earth’s atmosphere (ozone, oxygen, nitric oxides, water vapor, carbon dioxide, aerosols, etc.). These difficulties and accuracy of the SSI ground-based measurements are discussed by^1^.

SSI shows two different types of lines: absorption lines (named Fraunhofer lines after the scientist who discovered them) mainly above 150 nm, and emission lines below 150 nm. Both allow identification of the solar atmosphere’s chemical composition as well as the ionized atoms and their degree of ionization. Using the Doppler effect, the dynamics of the solar atmosphere can be observed, as well as the temperature as a function of altitude by measuring the line’s positions and shape, respectively. More recently, the solar diameter was measured as a function of wavelength on an absolute scale from space by using the Sun’s partial occultation by the Moon^2^. Such measurements allowed the validation of the solar opacity, which is a function of the composition, density, and temperature. These solar-diameter measurements are used in the validation of the model spectra^3^.

The thermal structure and composition of the Earth’s atmosphere depends on the radiation coming from the Sun. The power distribution of SSI has the capability to significantly affect the composition and the thermal structure of the middle atmosphere. Ultraviolet (UV) radiation acts on the atmospheric molecules and heats the middle atmosphere, as ozone warms the stratosphere. UV and extreme ultraviolet (EUV) light drive the ozone and atomic oxygen creation and destruction processes, leading to warming in the stratosphere and thermosphere, respectively. The concentrations of ozone and other atmospheric constituents therefore depend on SSI and its variability. Radiation at visible and near-infrared wavelengths reaches and warms the lower atmosphere and the Earth’s surface (ice/ocean/land), depending on its albedo. Gradients of temperature and density induce meridional and zonal circulation. The different atmospheric regions are also coupled by dynamical processes^4,5^. This brief summary shows the fundamental role of SSI and its variation on long-term climate forcing. The Sun, being a variable star, shows significant variations in SSI and TSI on a wide range of time scales from seconds to centuries. In order to improve the precision and accuracy of SSI and TSI measurements over time, successive missions have been planned by international space agencies from orbiters, NASA Shuttle missions, and rocket flights. In order to improve the accuracy of solar and terrestrial radiance and irradiance of absolute measurements, several agencies have initiated space radiometry traceability missions. European Space Agency (ESA) TRUTHS^6^, National Administration for Space and Aeronautics (NASA) CLARREO pathfinder^7^, and China Meteorological Administration (CMA) LIBRA^8^ are a few examples of such missions. All these missions require a comprehensive analysis of the existing SSI records.

Measurements from space experience different types of difficulties, especially for long-duration missions, due to the space environment. These effects include: optics damage by the space environment, outgassing, and high-energy particle effects on electronics, etc. These time dependent effects necessitate that an aging correction be determined and applied to the data products. In this article, we describe datasets from individual space missions, and also composite datasets created from several missions. The creation of a composite requires that one takes into account calibration uncertainties as well as uncertainties in the long-term trend corrections for each component dataset. For an SSI composite, the calibration and aging corrections are functions of wavelength, so the making of a composite can be a significant endeavor^9^.

Models of the solar spectrum and its variation come in two broad categories: theoretical and empirical. Theoretical models start with some estimates of the temperature structure of the solar atmosphere, then use principles of radiative transfer to compute the emergent spectrum. There is a wide range of such models with increasing sophistication. Current models not only use nonlocal thermodynamic equilibrium, but also magnetohydrodynamics to simulate flows in a magnetized plasma^10^. Another class of SSI model seeks to use a proxy (e.g., sunspot number) for changes in the solar atmosphere to create the corresponding spectrum^11^. The two most common models used in atmospheric and climate science are the Naval Research Laboratory SSI (NRLSSI)^12^ model and the Spectral and Total Irradiance REconstructions (SATIRE)^13^. NRLSSI uses the daily spectral measurements near 280 nm to represent the active regions (also known as faculae), and the sunspot area to represent darkening. A linear combination of these two components is used to model SSI at each wavelength. The model uses a daily image of the surface magnetism on the Sun to determine the relative contribution of each of several theoretical spectra. A more complete review of the range of models and observations can be found in a topical collection described in^14^.

**Fig. 1:**
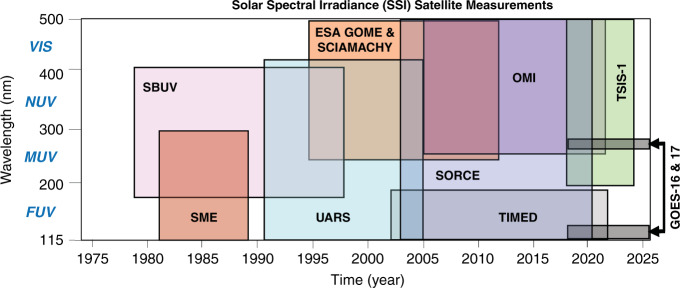
History of space-based daily observations of SSI in the ultraviolet- and visible-wavelength domain. In addition, several rockets flights and Shuttle missions were made for different aims of calibration and validation. The SOLAR platform composed of three instruments (SOLSPEC, SolACES, and SOVIM) was placed on the International Space Station and operated from 2008 to 2017. SOLAR measured the SSI from the EUV to near IR (3000 nm), Courtesy of Tom Woods (LASP)

The ensemble of successive space missions has, over time, improved our knowledge of the solar spectrum. The state of the art of SSI measurements is summarized here. Figure 1 shows the history of space-based daily observations of SSI in the ultraviolet and visible wavelength domains. The far ultraviolet (FUV, 115–200 nm) range has been regularly observed since the early 1980’s with only a small gap between the Solar Mesosphere Explorer (SME^15^) and the Upper Atmosphere Research Satellite (UARS^16^) missions. With the end of the Solar Radiation and Climate Experiment (SORCE^17^) mission in January 2020, the only available FUV data now come from the Thermosphere Ionosphere Mesosphere Energetics and Dynamics (TIMED^18^) mission. When TIMED (launched in 2002) ends, there will be a gap in FUV observations. At longer ultraviolet wavelengths, there is measurement continuity with the Total and Spectral Irradiance Sensors (TSIS-1^19^) mission currently producing data from 200 nm to the near-infrared. Visible-light observations began in the mid-1990’s with the ESA Global Ozone Monitoring Experiment (GOME-1^20^) and continue with several overlapping datasets.

The requirements for calibration and aging corrections vary from one wavelength domain to another. As will be discussed later in this paper, SSI variability changes greatly from one wavelength domain to another. At the shortest wavelengths, the Sun varies by a factor of two or more over the solar cycle, while the variation in the visible range is a fraction of a percent. Aging corrections must be precise enough to capture this variation.

Accurate SSI and TSI measurements contribute to advancement in the following disciplines.

## In solar physics

Solar-irradiance observations are used for validating theoretical solar models^21,22^. Irradiance measures global changes in the Sun’s output and serves as an important constraint on global dynamo models. We note that this validation is limited by the accuracy of the measurements. Measurement uncertainties are a combination of preflight calibration uncertainty as well as uncertainty in making a correction for aging in space. This is why successive as well as redundant missions and rocket flights are necessary for validating and improving the accuracy of the measurements.

## In planetary atmosphere physics

Understanding of planetary-atmosphere data requires SSI data^23,24^. It is the most convenient when both planetary atmosphere and solar data exist at the same time. However, in many situations, there are no simultaneous observations. In that case, solar models are needed to generate SSI at the appropriate time in the appropriate spectral range. Furthermore, for predicting the evolution of a planetary atmosphere, theoretical, semiempirical, or reconstruction solar models are often necessary given the long-time scales.

## In Earth’s climate physics

In the case of climate modeling of past periods, SSI reconstructions are needed since no solar measurements exist. Solar-radiative forcing is the largest input to the Earth’s climate system, about 10^4^ larger than all other inputs combined^25^. Even small changes in irradiance levels can have profound impacts on the Earth’s radiative budget^23,24,26–28^. For example, the Maunder (1645–1710) and Dalton (1807–1810) minima are critical periods in climate history, but no SSI observations are available. For these past periods, indirect information related to solar activity is used. We classify this information into two categories: historical records and modern measurements of quantities that are correlated to solar activity. The first category includes sunspot number: the 11 year solar-cycle length, the aurora’s frequency and latitude of their appearance, etc. The second category includes carbon dating of tree rings and radioisotope ratios extracted from ice cores^29^. Both categories are called proxies and constitute a primary source of information for SSI reconstructions, which are afterward employed in historical climate studies.

The results in terms of climate and atmosphere simulations are dependent on SSI models. Choosing the right SSI model is critical. Several examples illustrate the influence of the SSI^4,5,30–33^. It is particularly difficult to simulate low-activity periods such as the Maunder and Dalton minima. All the modern data come from an epoch of greater solar activity, the so-called Modern Maximum^34^. This is why the recent minimum occurring in 2008 is particularly important for solar, atmosphere, and climate physics. This minimum occurred about two years later than the standard solar-activity predictions. The average length of a solar cycle is 11 years, but cycle 23 was closer to 13 years. The 2008 activity level is used in climate models to represent a lower activity level than has been seen for many cycles. By comparing successive SSI minima, we can also estimate the magnitude of any secular trend in solar activity. Measuring a secular trend requires detailed understanding of the calibration and aging corrections of missions/composites over several decades. Estimating the accuracy of theoretical and empirical models as well as observations is the primary objective of this article.

For this study, data for solar cycles 23 and 24, measured from space by spectrometers, theoretically modeled, or provided by semiempirical models, will be used. Table 1 lists the wavelength coverage for each measurement or model considered in this paper. This set will allow us to compare

- The time-series variability at some specific wavelengths,
- The measurements and their predictions by theoretical and semi-empirical modeling for the 2008 solar minima,
- The spectral measurements and their predictions by theoretical and semi-empirical modeling in the absolute scale,
- The derived brightness temperature as a function of wavelength.
  The spectra used in this study are:
- ATLAS3^35^ SOLSPEC^36^ is a triple dual spectrometer for UV, visible, and near-IR up to 2400 nm. It was equipped with lamps to verify the stability of the instrument. It flew on the NASA Space Shuttle with SL1 (1983), ATLAS1, 2, 3 in 1992, 1993, and 1994, respectively for one-week duration, and on the EURECA platform for 6 months starting in 1992.
- PREcision MOnitoring Sensor (PREMOS^37,38^) onboard the PICARD satellite. The PREMOS experiment consisted of five photometers with bandpasses in the 210 nm, 266 nm, 535 nm, 607 nm, and 782 nm regions of the spectrum. The PICARD mission was active between 2010 and 2014.
- NLTE Spectral SYnthesis code (NESSY^39^): a theoretical model, which is an improvement of the former COde for Spectral Irradiance (COSI^40^).
- Spectral and Total Irradiance REconstructions (SATIRE^13^): a semiempirical model. SATIRE uses daily magnetograms to determine the relative contribution of magnetic-field structures on the solar disk. A linear combination of solar atmospheric models then produces the daily irradiance spectrum.
- The First European SOLar Irradiance Data Exploitation (SOLID^41^): a composite model. SOLID is based on all of the available observed time series. It combines them in a weighted average that tries to maximize the information content of the dataset.
- SOLAR-ISS Rev1 using SOLar SPECtrum (SOLSPEC^1,36,42^) and Solar Auto-Calibrating EUV Spectrometer (SolACES^43^) instruments onboard ISS. SOLSPEC is a dual monochromator with heritage from the previous SOLSPEC instrument^36^ and measures from 175–2400 nm. SolACES is an ionization chamber spectrometer that measures in the EUV (16.5 nm–140 nm).
- SOLAR3^44^ based on reprocessed SOLSPEC-ISS measurements.
- SOlar Radiation and Climate Experiment (SORCE^17^): EUV to IR daily average irradiances. X-ray Photometer System^45^ version 11, SOLar-Stellar Irradiance Comparison Experiment^45^ version 17, and Spectral Irradiance Monitor^46^ version 25 are used in this study. XPS observes discrete bandpasses in the EUV, SOLSTICE data cover the 115–300 nm range, and SIM observations are used from 300 to 2400 nm. SORCE data were downloaded in January 2020. The SORCE web page provides a single daily spectrum combining measurements from these three instruments. The version downloaded in 2019 will be referred to as V16, while the dataset downloaded in 2020 will be V17. Each of the three components has its own version number, but we will use only the SOLSTICE versioning for simplicity.

**Table 1:** Wavelength domains (*λ*_1_ to *λ*_2_) per spectra used for this study, and their origin

Name PREMOS^a^ NESSY^b^	SATIRE^b^	SOLID^c^	SOLARrev^a^	SOLAR3^a^	SORCE^a^	ATLAS3^c^
*λ*_1_ Set of 5 90	115	0.5	16.5	0.5	0.5	0.5
*λ*_2_ wavelengths 2400	2400	1900	2400	2400	2400	2400

^a^Measurements

^b^Theoretical or empirical model

^c^Composite of measurements from multiple instruments

Theoretical and semiempirical composite models have the capability to be run at different time periods. Time coverage from space measurements are obviously linked to the missions’ operations. For this study, data for solar cycles 23 and 24 will be used. As SORCE has presently the longest time series, we have focused our study on these measurements.

To intercompare these spectra, we have used ATLAS3^35^, which is an independent composite model using data from the three ATLAS Shuttle missions, EURECA platform, and rockets flights, obtained during the previous minimum (cycle 22–23). This choice is based on Shuttle missions lasting a week and using laboratory calibration before and after each flight. The SORCE and the model spectra have the same wavelength range as ATLAS3.

## Results

### In time series

**Fig. 2:**
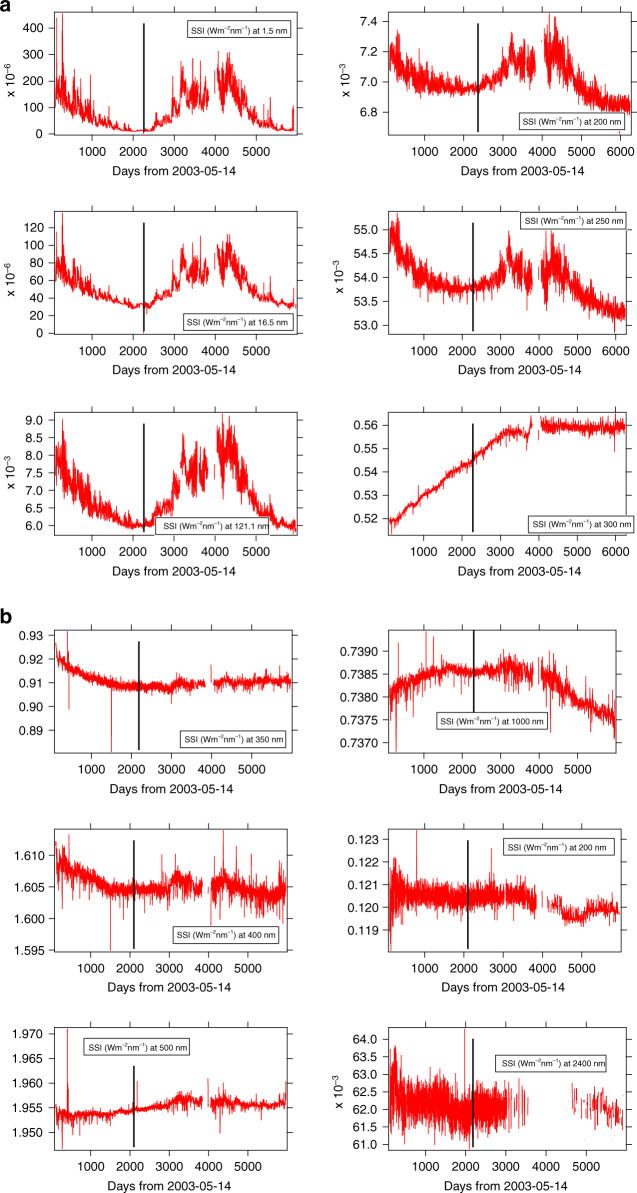
Time series observed by SORCE v17 for 6 wavelengths. **a** 1.5–300 nm, **b** 350–2400 nm. The black bar indicates the 10 January 2009 for which a minimum appears in the EUV range

The composite SORCE SSI data product, downloaded from the LISIRD web page (http://lasp.colorado.edu/lisird) in January 2020, was used in this analysis. Figure 2 shows time series from instruments aboard SORCE^17^ for selected wavelengths from the extreme ultraviolet to the infrared. The selected wavelengths are 1.5 nm, 16.5 nm, Ly *α* (Lyman *α* at 121 nm), 200 nm, 250 nm, 300 nm, 350 nm, 400 nm, 500 nm, 1000 nm, 2000nm, and 2400 nm. One important feature to notice is an earlier date of minimum in the EUV than at longer wavelengths. Up to 250 nm, a clear solar minimum is shown on 10 January 2009 (within a few days). However, in the EUV, the minimum found by SolACES^47^ is a few months earlier (August 2008). The early 2009 date of minimum is roughly consistent with the study by^48^ that found a date of late 2008 for the minimum in TSI, Mg II, and F10.7.

At 250 nm, the SSI decrease from the start of the SORCE mission is about 2%. SORCE began measuring in mid-2003, which was a few years after the solar cycle 23 maximum. At 300 nm, no clear minimum is displayed; however, a 2% amplitude variation is shown, which is not in phase with the SSI at the other wavelengths. Likely, this variation is of instrumental origin. Above 300 nm, SSI variations are from instrumental origin rather than solar origin^17^.

**Fig. 3:**
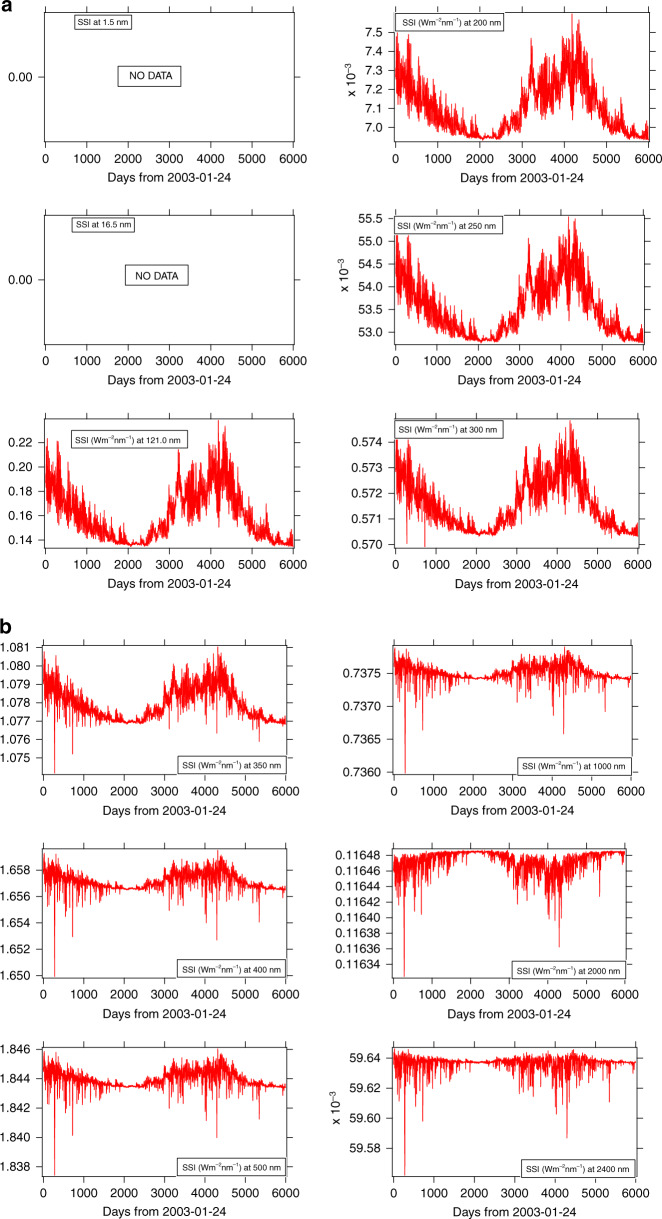
Modeled time series by using SATIRE. **a** For 10 wavelengths from EUV to IR, 121.0–300 nm. **b** For 10 wavelengths from 350–2400 nm

We also compared SSI time series between models and observations. Figure 3a, b shows the UV-to-IR SSI time series modeled by SATIRE. The SATIRE results show SSI variation in phase with SORCE measurements up to 1000 nm with decreasing amplitude, so small that above 300 nm, SORCE/SIM cannot measure it. The uncertainty in the solar cycle behavior of SORCE/SIM has been a lively topic of debate, and there have been several investigations to reanalyze SORCE/SIM data^17,49–51^ to improve the aging correction and extract the solar-cycle signal. Interestingly, while a minimum was shown below 1000 nm in January 2009, there is a maximum around 1000 nm which disappears by 2000 nm^52^. As a function of wavelength, the minimum observed on 10 January 2009 has a decreasing amplitude from 30% at Ly *α* to 0.5% at 300 nm, explaining why such very small variation is masked within the measurement noise and possible long-term drift due to uncertainty in the aging correction of SORCE/SIM.

**Fig. 4:**
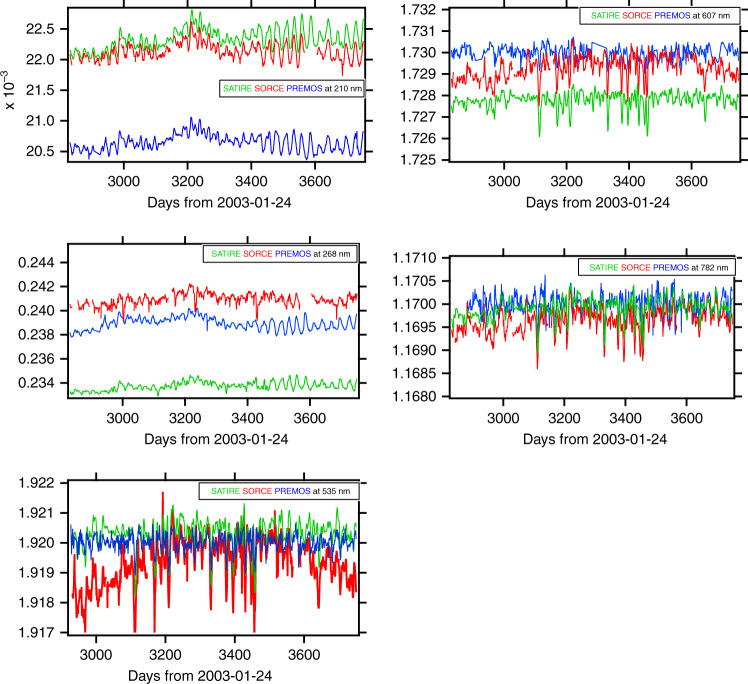
Time series measured by PREMOS and SORCE v17 and SATIRE model during the PICARD. These datasets are compared at 210, 268, 535, 607 and 782 nm wavelength. The date format is the same as for Figs. 2 and 3

Figure 4 shows the middle UV to near IR in specific spectral domains (210 nm, 268 nm, 535 nm, 607 nm, and 782 nm) measured by PREMOS sun-photometers onboard the PICARD spacecraft, together with SORCE measurements and SATIRE model results at those wavelengths. The comparison shows data from July 2010 to April 2014 (end of PICARD mission). During this four-year time period, the Sun went from low to moderately high activity with the onset of solar cycle 24 (Fig. 4).

PREMOS channels at 210 nm and 268 nm are calibrated in absolute units. At 535 nm, 607 nm, and 782 nm, data are given in arbitrary relative units. Nevertheless, they are displayed with the two other time series (SORCE and SATIRE) for comparing their variability.

We point out the following features:

- at 210 nm and 268 nm, the SSI’s variability is highly correlated without significant time shift.
- from days 3400 to 3750, the five UV channels of PREMOS are correlated showing synchronous variations due to the solar rotation (see^38^ for more detailed comments).
- In the visible and near-infrared, the maximum at about day 3200 (29 October, 2011) observed in UV has disappeared, and a very small SSI decrease may be seen on these three spectra, very likely due to the transit of some sunspots. Variability at these wavelengths is very small, about 0.1%. SORCE variability is greater than predicted by SATIRE as well as measured by PREMOS.

The correlation coefficients calculated for these time series are shown in Table 2. This is one way to quantify the agreement between these spectra. The coefficients are estimated by using the robust correlation analysis^47,53^, which computes the conditional means and variances to test variance homogeneity based on a percentile bootstrap with adjustment for small samples. It outputs the 95% confidence intervals (CI) and the histogram of the bootstrapped estimates, which eliminates any bias due to outliers.

**Table 2:** Correlation coefficients between the SATIRE, SORCE-V16, SORCE-V17 and PREMOS during the PICARD operations (August 2010–April 2014)

Wavelength (nm)	210	268	535	607	782
SATIRE/SORCE V16	0.64	0.56	0.47	0.42	0.48
SATIRE/PREMOS	0.84	0.71	0.63	0.53	0.52
SORCE V16/PREMOS	0.89	0.60	0.43	0.41	0.40
SATIRE/SORCE V17	0.69	0.58	0.47	0.43	0.49
SORCE V17/PREMOS	0.81	0.57	0.44	0.39	0.41

We computed the correlation coefficients for two different versions of the SORCE-combined daily irradiance, v16, and v17. Version 17 had an update to the aging corrections for SOLSTICE (210 nm and 268 nm) and SIM (535 nm, 607 nm, and 782 nm). It was published in late 2019. This data version became available after the original presentation of this analysis at the LIGHT conference in July 2019. The differences between the two SOLSTICE versions give an indication of the uncertainty in the aging correction for that dataset. We note the high agreement between PREMOS and SATIRE, as well as PREMOS and SORCE/SOLSTICE V16, especially in the UV. However, the correlation decreases in visible and near-IR, likely due to an instrument drift responsivity with time, the smaller measurement number at 607 nm, and the quasi absence of periodic variations in these spectral domains.

We note a better agreement between SORCE/SOLSTICE V17 and SATIRE from UV to IR, than with V16, however, an opposite conclusion appears from the comparison between SORCE/SOLSTICE and PREMOS. We note that for this comparison, data from August 2010 to March 2014 are used covering the period (days 3400 to 3750) during which the two instruments made simultaneous observations of the solar-rotation effect (see Fig. 4). We note that during this period^45^, solar rotations were observed, allowing reduction of noise, despite the fact that the solar-rotation effect is only about 1%. In addition, the UV SSI V17 and SATIRE are closer than SATIRE and V16.

- PREMOS operated during 4 years of moderate solar activity and mainly observed the sunspots’ rotational effect on the variation of SSI with a correlation coefficient around 0.89 for SORCE/SOLSTICE V16 and 0.81 for SORCE/SOLSTICE V17.
- At the opposite end of the activity timescale, SORCE operated for over 6000 days, which allowed measurement of the 11-year solar-cycle effect. SORCE/SOLSTICE SSI long-term variation from 2003 to 2008 is smaller for V17 than for V16 (2 and 6%, respectively). This solar-cycle variability is in better agreement with models and other measurements^54^ (see Fig. 8). The correlation between SATIRE and SORCE is roughly the same for the two data versions. The two datasets are in agreement on the amplitude of the SSI rotational variation.

**Fig. 5:**
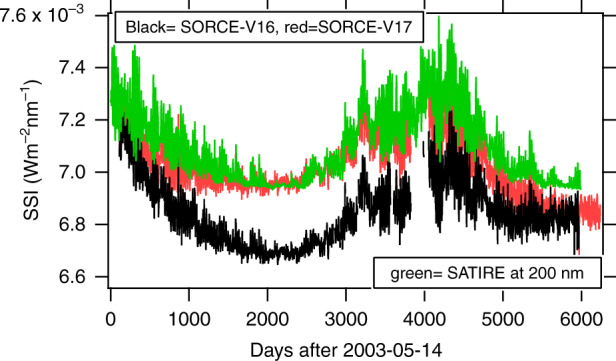
Comparison between time series measured by SORCE V16, V17, and SATIRE at 200 nm

SORCE/SOLSTICE SSI changes to the aging correction in V17. In particular, the calibration measurements in the last six years of the mission were used and trends were interpolated to update the degradation estimate for the entire mission. Further analysis is ongoing and a new SOLSTICE version (V18) was released in late 2020 (release notes and ATBD references: https://scholar.colorado.edu/concern/reports/p8418p20s (SOLSTICE RELEASE NOTES) https://scholar.colorado.edu/concern/reports/rx913r207(SOLSTICE ATBD)). An important result, which is relevant to this article, is that the magnitude of solar cycle 23 was greatly reduced in V17. This has brought the SOLSTICE SSI measurements into better agreement with several models such as NRLSSI2^54^. Owever, the trends in SSI during the decline of solar cycle 24 (2014-2020) are larger than expected. This is shown in Fig. 5. We also add that Shannon’s criteria, which requires twice the 11-year solar cycle, duriation of measurements, is not exactly met.

### In comparison of the absolute SSI as a function of wavelength

**Fig. 6:**
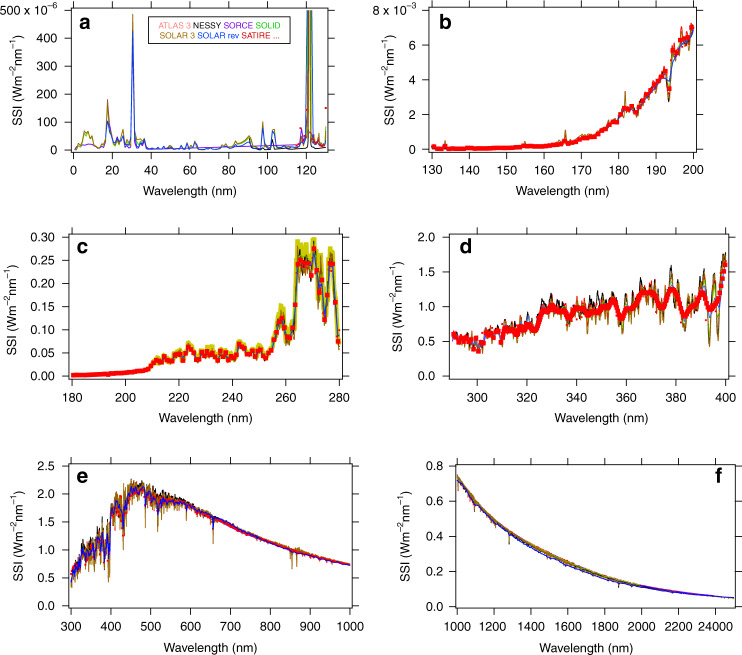
Seven spectra around solar-activity minimum. **a** Seven spectra in the domain 0–130 nm. This wavelength range shows intense emission lines (e.g. He line at 30.5 nm), **b** seven spectra in the domain 130–200 nm. This wavelength range shows the transition from emission to Fraunhofer lines. **c** Seven spectra in the domain 180–280 nm. Above 200 nm, SSI shows many Fraunhofer lines, in which depths decrease as the wavelength increases (see d–f). **d** Seven spectra in the domain 280–400 nm. **e** Seven spectra in the domain 400–1000 nm. **f** Seven spectra in the domain 1000–2400 nm

**Fig. 7:**
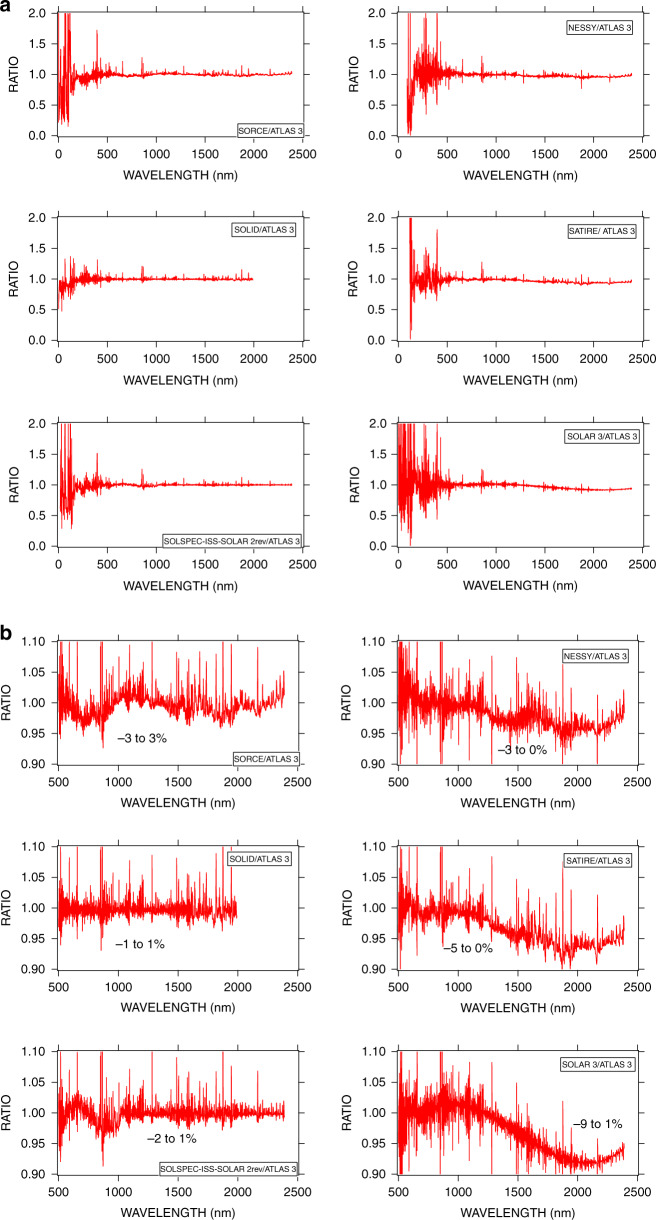
Ratio of the six spectra to ATLAS3. **a** From 0 to 2400 nm with a ratio scale from 0–2. **b** Above 500 nm with an expanded ratio scale from 0 to 1.1

**Fig. 8:**
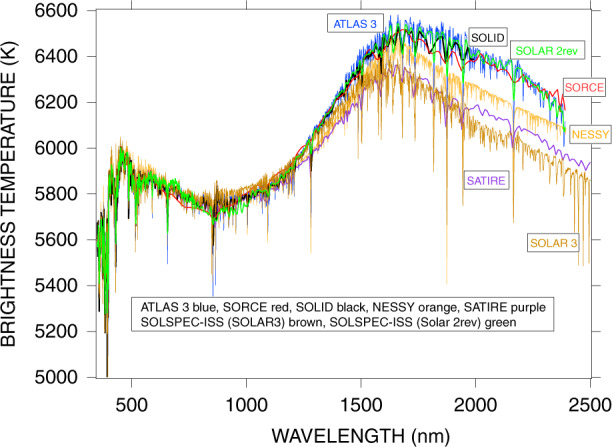
Brightness temperature in the visible-to-IR range for the seven spectra used in this study

Four different approaches are used to compare absolute SSI:

- High spectral-resolution spectra (Fig. 6).
- SSI ratio to a given spectrum (Fig. 7).
- Power in large-wavelength ranges to avoid the effects of having spectra based on data from spectrometers of different band-pass shapes (Table 3).
- Brightness temperature (BT), which is a physically meaningful way to show SSI differences, especially in the IR domain (Fig. 8).

The spectra displayed in Fig. 6a–f correspond to a time period around minimum solar activity, which was comparable to ATLAS3, SOLAR rev, SOLID, and SOLAR3. SORCE data for this period were V17 for SOLSTICE and V25 for SIM. The results from NESSY and SATIRE were calculated for this period (April 2008).

Figure 6a shows 7 spectra from 0 nm to 130 nm, however, SATIRE results are only represented in the domain 115–130 nm as SATIRE does not provide data below 115 nm (see Table 1). Around 5 nm, ATLAS3^35^ and SOLID agree, while SOLAR3 and SORCE (XPS and SOLSTICE) also agree but at a lower value. Up to 80 nm, there is no significant disagreement, except at 18 nm, due to SolACES included in SOLAR rev. Above 80 nm, instrumental band-pass shifts and/or shape are likely explaining the difference in measured emission lines.

Figure 6b shows 7 spectra from 130 nm to 200 nm. There are no significant discrepancies. Most of the differences are due to Fraunhofer and emission lines measured by different instruments having different spectral resolution.

Figure 6c shows the 7 spectra from 200 nm to 280 nm. There are no significant differences. SOLID shows a SSI higher than the others. This point will be numerically estimated in Table 3.

**Table 3:** Power in *Wm*^*−*2^ per spectral domains

Spectrum	0–	115–	290–	400–	115–	400–	115–	1000–
Range (nm)	90	290	400	1000	1900	1900	2390	2390
(1) ATLAS3	0.002534	9.9667	101.04	847.830	1281.71	1171.84	1329.67	372.32
(2) SOLARrev	N.A.	9.5005	99.819	845.625	1277.57	1169.55	1325.73	372.239
(3) SOLAR3.	0.002560	9.6610	98.2505	851.015	1276.77	1170.01	1321.02	363.618
(4) SORCE	0.001774	9.8800	99.38	841.105	1273.10	1166.21	1320.91	373.213
(5) SOLID	0.002234	9.9009	100.728	845.670	1278.32	1168.91	N.A.	N.A.
(6) SATIRE	N.A.	9.4630	98.978	839.480	1272.98	1165.63	1306.73	360.275
(7) NESSY	N.A.	9.9490	103.877	855.420	1286.80	1174.09	1332.94	359.109
*µ*	0.002270	9.7600	100.29	846.590	1278.18	1169.46	1322.83	366.79
*σ*	0.000316	0.2000	1.71	5.10	4.50	2.75	8.40	5.95
*σ/µ* (%)	14	2.0400	1.70	0.60	0.35	0.24	0.63	1.6

Among the seven spectra, for some of them not having the same wavelength coverage as the others, the mean cannot be calculated in certain ranges, indicated by N.A. (e.g., SATIRE between 0 nm and 90 nm, see Table 1)

Figure 6d shows the 7 spectra from 280 nm to 400 nm. The Fraunhofer lines are well shown by these spectra. All of the datasets shown in this panel agree within their uncertainties.

Figures 6e, f display data from 400 nm to 1000 nm and 1000 nm to 2400 nm, respectively. The differences are small and will be described in the following two sections.

SSI ratio of each spectrum to ATLAS3 as a function of wavelength for several spectral domains. We have chosen ATLAS3 as the common denominator for the following reasons:

- It is an independent measurement (except SOLID in the IR domain).
- SOLSPEC made measurements onboard the three ATLAS missions, which each have a duration of a week. These short flights do not require a significant aging correction. Furthermore, for each mission we had a postflight recalibration. The IR part of ATLAS3 spectrum was measured first time from space onboard the EURECA platform^55^ and confirmed in 2008 with SOLSPEC onboard the ISS^1^.
- Before flight, SOLSPEC has been calibrated in the absolute scale. It has an absolute preflight calibration using the Physikalisch-Technische Bundesanstalt (PTB) blackbody. PTB is independent from the NIST facility used in the SORCE/SOLSTICE calibration. This is an important point: if two independently absolutely calibrated datasets are in agreement, it is unlikely that either one suffers from significant systematic errors in calibration. ATLAS3 overlaps SORCE measurements up to 2400 nm. The UARS SSI observations were not used in this study because data from UARS/SOLSTICE and UARS/SUSIM do not go beyond 410 nm.

Figure 7a shows that the EUV and UV regions present large oscillations of the ratios for all spectra. Absolute calibration of the instruments cannot fully explain such a result. It is mainly due to the instruments’ band passes being either slightly shifted relative to each other or having slightly different shapes (rectangular or of Gaussian type), which generate different signals in the presence of emission lines. A typical case would be a line placed at the center of the bandpass for one instrument, while it might be in the wings for another one, as suggested by Fig. 7a.

Above 500 nm, the Fraunhofer line density and its depth decrease as compared with the UV domain. These ratios have significantly decreased as shown by Fig. 7b with an expanded scale.

The best agreement with ATLAS3 is found for SOLID, due to the method of construction of this spectrum. This is explained in^41^ by a normalization of the SOLID composite spectrum and an adjustment to the solar activity using the TSI (see section 3.7 of the cited above article).

The SORCE and SOLSPEC-ISS SOLAR rev show an agreement within 3% (left panels of Fig. 4b), which is consistent with their own accuracy. As for its right panel, a decrease in the IR is present for theoretical, composite, and SOLAR3. For the latter, a difference of 9% is reached at 2000nm and above. This point will be discussed in item 4.

Power differences between spectra in several wavelength domains.

Figure 7a, b shows that existing differences are a function of wavelength. However, absolute calibration accuracy, as well as band-pass difference (shape and position), both show effects at a few-nm resolutions. This is why we show SSI comparisons over large spectral domains. In order to investigate the power difference between the seven spectra, we have integrated the spectra in different large spectral domains, where *μ* is the average value and *σ* is the standard deviation for a given wavelength range, as shown in Table 3:

- In the range 0–90 nm, the power ratio *σ/μ* reaches 14% despite the large domain of integration. Above this wavelength, the ratio decreases due to the decreasing number of spectrally resolved lines in the spectrum. Fewer spectral features decrease σ without changing µ. SOLAR3 and ATLAS3 are in very good agreement below 1000 nm.
- In the range 115–290 nm, the power dispersion is 0.2 Wm^*−*2^, meaning a high consistency of the spectra.
- In the range 290–400 nm, and up to the 400–1900 nm domain, the *σ/μ* remains below 1%. It increases above due to the discrepancy of the spectra in the IR SSI.

As for the V17–V16 difference, we have considered two significant domains 115–290 nm and 290–400 nm (see Table 4).

**Table 4:** Comparison of the SORCE V16 with V17 power in two specific wavelength domains

Range (nm)	115–290	290–400
SORCE V16 (Wm^−2^)	9.16	97.82
SORCE V17 (Wm^−2^)	9.88	99.38
(V17-V16)/V16	7.8%	1.6%

The difference increases of 7.8% in the range 115–290 nm and 1.6% in the range 290–400 nm as expected, since the primary change was in the SOLSTICE data.

We note a higher power and a better *σ* /*μ* in the UV when using V17 with respect to the results using V16. This is consistent with the changes made in V16.

On comparison of the brightness temperature derived from the selected spectra, it is calculated from the Planck function

1 $${\it{L}}(\lambda ,T)=2hc^2\lambda ^{-5}/(exp(hc/\lambda kT)-1)$$

where T is the temperature, and L is the luminosity. *λ* is the wavelength, c, k, and h are the light velocity, the Boltzman constant, and the Planck constant, respectively. L is derived from SSI/Ω, where Ω is the solid angle of the Sun as seen from the Earth at one AU, which is calculated from its radius. Given the uncertainty in the solar radius^2^, we have derived the brightness temperature at 600 nm for two radius values (959 and 969 arcseconds). The temperature changed by about 20 K, which is negligible, compared with the noise in the daily irradiance measurements. Following the analysis in^2^, we have adopted 959.75 arcseconds as the mean value for the solar radius^2^.

Figure 8 shows the brightness temperature derived from the 7 spectra. Brightness temperature emphasizes small SSI differences between spectra, especially for longer wavelengths. A peak of brightness temperature (BT) at 1600nm is calculated for all 7 datasets: theoretical models, semiempirical models, and measurements. BT as a function of wavelength is distributed in three families. They are:

- The upper one with ATLAS3, SOLID, SORCE, and SOLSPEC rev. These are all essentially measurements, and the BT differences between them are not larger than 100 K.
- At about 140 K lower temperature, there are the NESSY and SATIRE models. They have the same shape and a temperature difference not larger than 100 K. We note that in the IR, the temperature dependence with wavelength is quasi-linear.
- The lowest BT distribution is given by SOLAR3. In the IR domain, the black body emission is approximated by the Rayleigh–Jeans law as T /λ^4^. The lower BT is a consequence of the lower SSI at wavelengths longer than 1000 nm relative to all other spectra. Discussion of the source for this lower irradiance is described in^56^. At 2400 nm, BT is about 200 K below the NESSY temperature, which is consistent with the temperature distribution of the photosphere^57^.

### In proxies for solar-activity modeling

The first category of proxies has been described in the climate physics section. A second category concerns the solar magnetic field and spectroscopic measurements initiated during the 20th century, such as the Mg-II core to wing ratio (280 nm), Ca-II (393 nm) core to wing ratio, and the F10.7 cm radio-flux index. Variations in these measurements have a high correlation with solar variability at other wavelengths^58^.

The use of the solar-activity proxies permits one to reconstruct the SSI when no direct measurement exists. The Ly *α* emission line, and Mg II and Ca II lines are proxies that are frequently used in modeling^56,57^^,^^59–61^. These lines have some specific characteristics explaining why they are used. The Ly *α* emission line is very intense (see Fig. 4a) and is formed over a wide range of heights in the solar atmosphere. The Mg II and Ca II proxies are produced from ratios of adjacent spectral regions, so they do not need to have an aging correction^56,57^.

Since SORCE provides the longest-measurement time series, it provides a unique opportunity to compare the performance of an empirical model such as SATIRE over more than one solar cycle. The SSI variability reconstructed by SATIRE is in good agreement with SORCE measurements over the 17-year mission, i.e., correlation coefficients above 0.80 for both V16 and V17 and for Ly α and the Mg II index (Table 5). However, we note that V17 provides a higher correlation with SATIRE than V16, in particular at 390 nm by either a noise reduction or an improvement in the long-term drift or both. Furthermore, the correlation coefficients decrease from Ly *α* to Ca II. Ca II has a lower contrast relative to the continuum. The SATIRE irradiances are independent of the proxies since they depend only on magnetograms.

**Table 5:** Correlation coefficients between the SATIRE and SORCE time series for Ly *α*, Mg II, and Ca II lines as solar activity proxies

Wavelength	Ly*α* (121 nm)	Mg II (280 nm)	Ca II (390nm)
SATIRE/SORCE-V16	0.9440	0.8879	0.3938
SATIRE/SORCE-V17	0.9759	0.8960	0.5971

As for the Mg II emission, the chromospheric lines are located in the core of a Fraunhofer absorption line, an index of variability is defined as the core-to-wing ratio^57^. The correlation coefficient (above 0.90) between the two time series shows the capability of this index to accurately represent the solar variability. Further studies have shown that this index is also highly correlated with EUV variability.

The Ca II Fraunhofer lines work in a similar fashion as Mg II. However, the Ca II lines have a smaller intensity and a smaller variability than the Mg II chromospheric lines^60^. Therefore, the noise affecting the measurements becomes important for extracting the variability in Ca II and explains the lower value of the correlation coefficients.

Consequently, V17 provides higher correlation coefficients than V16, in the UV domain. However, this is not observed in the visible-to-near-IR, meaning that if solar cycle variability exists at these wavelengths, it is likely below the uncertainty in the aging correction.

### In the solar activity at the transition between solar cycle 23–24

The 2008 minimum, likely lower than the other recent minima^62^ (cycles 21–22 and 22–23) offers an important opportunity for this study. However, as minimum SSI is used by several reconstructions, and as the surface magnetic features, sunspots, are obviously less apparent at solar minimum activity than at solar maximum, the model predictions have a larger relative uncertainty. This is why it is important to point out the differences existing among the available spectra for solar minimum conditions.

Not all measurements showed the same date for the transition from cycle 23 to cycle 24. Depending on the nature of the data (TSI, SSI, and solar proxies), different times for solar minimum were found. This is consistent with the results of^48^ . They found three different times for the minimum of TSI, F10.7, and the Mg II index from SORCE/SOLSTICE. For SORCE/TIM TSI, it was spring 2009. Between January 2008 and June 2009, the Mg II index presents a wide minimum centered on June 2008.

The EUV spectrum observed by the SolACES spectrometer onboard the ISS measured the integrated EUV SSI from 16.5 nm to 29 nm^63^. Showed a EUV minimum occurring on 21 August 2008. The data presented in Fig. 2 show the time of minimum to be 10 January 2009 for EUV–UV wavelengths. The disagreement between SolACES and SORCE EUV measurements is currently unresolved and will need analysis beyond the scope of the current study. Table 6 summarizes the results from the different datasets we have used.

**Table 6:** Date of minima provided by the data used in this study

Sources	Date
TSI	Spring, 2009
16.5 to 29 nm (SolACES)	21 August 2008
1.5 nm to 250 nm (SORCE)	10 January 2009
Mg II	June 2008

## Discussion and conclusion

Three types of recent SSI measurements, semi-empirical models, and radiative transfer models, extending from EUV to IR or UV to IR, have been examined. Comparisons presented here used 7 spectral datasets. They show:

- In the visible and IR, it is noticeable that 6 of the 7 spectra agree within a few percent.
- In the UV and EUV, there are some disagreements, which are due to the different absolute calibration, pass-band profiles, and centering. The latter two aspects being the most important in wavelength regions containing emission and Fraunhofer lines.
- Intercomparison of SSI models for reconstructions is summarized in several recent papers^63,64^. As mentioned in^2^, improvements in theoretical solar models will largely depend on improved laboratory atomic data such as the Vienna Atomic Line Database^65^. Furthermore, the pass-band profile and centering are frequently different for different instruments and also subject to aging. Today, their monitoring remains a challenge. The SSI also allows derivation of the brightness temperature using the Planck function. The 7 spectra used here all produce a maximum temperature close to 1600nm. However, the value of the temperature is higher for observational data than for semiempirical and radiative-transfer models by about 200 K. About SSI variability, the solar-rotation effect measured by SORCE and PREMOS–PICARD accurately agrees. However, the long-term variability remains a difficult task due to the instruments aging in the harsh space environment. We conclude that there is a convergence between theoretical, semiempirical approach, and measurements within their individual uncertainty envelopes, and improvements are anticipated.

However, some differences do exist between the SSI models whatever their origin likely generated by component aging in space environment due to particles, EUV and UV, and contaminant deposition. Significant improvements should occur by using more robust detectors and optics, and low outgassing components as technologies are always moving forward.
